# Lewis y antigen promotes the proliferation of ovarian carcinoma-derived RMG-I cells through the PI3K/Akt signaling pathway

**DOI:** 10.1186/1756-9966-28-154

**Published:** 2009-12-15

**Authors:** Juanjuan Liu, Bei Lin, Yingying Hao, Yue Qi, Liancheng Zhu, Feifei Li, Dawo Liu, Jianping Cong, Shulan Zhang, Masao Iwamori

**Affiliations:** 1 Department of Obstetrics and Gynecology, China Medical University Shengjing, Hospital, 36 Sanhao Street, Heping, Shenyang, 110004, PR China; 2 Department of Biochemistry, Faculty of Science and Technology, Kinki University, 3-4-1 Kowakae, Higashiosaka, Osaka, 577-8502, Japan

## Abstract

**Background:**

Lewis y antigen is difucosylated oligosaccharide and is carried by glycoconjugates at cell surface. Elevated expression of Lewis y has been found in 75% of ovarian tumor, and the high expression level is correlated to the tumor's pathological staging and prognosis. This study was to investigate the effect and the possible mechanism of Lewis y on the proliferation of human ovarian cancer cells.

**Methods:**

We constructed a plasmid encoding α1,2-fucosyltransferase (α1,2-FT) gene and then transfected it into ovarian carcinoma-derived RMG-I cells with lowest Lewis y antigen expression level. Effect of Lewis y on cell proliferation was assessed after transfection. Changes in cell survival and signal transduction were evaluated after α-L-fucosidase, anti-Lewis y antibody and phosphatidylinositol 3-kinase (PI3K) inhibitor treatment.

**Results:**

Our results showed that the levels of α1,2-FT gene and Lewis y increased significantly after transfection. The cell proliferation of ovarian carcinoma-derived RMG-I cells sped up as the Lewis y antigen was increased. Both of α-L-fucosidase and anti-Lewis y antibody inhibited the cell proliferation. The phosphorylation level of Akt was apparently elevated in Lewis y-overexpressing cells and the inhibitor of PI3K, LY294002, dramatically inhibited the growth of Lewis y-overexpressing cells. In addition, the phosphorylation intensity and difference in phosphorylation intensity between cells with different expression of α1,2-FT were attenuated significantly by the monoantibody to Lewis y and by the PI3K inhibitor LY294002.

**Conclusions:**

Increased expression of Lewis y antigen plays an important role in promoting cell proliferation through activating PI3K/Akt signaling pathway in ovarian carcinoma-derived RMG-I cells. Inhibition of Lewis y expression may provide a new therapeutic approach for Lewis y positive ovarian cancer.

## Background

Lewis y antigen is carried by glycoconjugates (glycoproteins and glycolipids) at cell surface. It is an oligosaccharide with two fucoses, and its chemical structure is Fucα1 → 2Galβ1 → 4 [Fucα1 → 3]GlcNAcβ1 → R, belonging to the A, B, H, Lewis blood group antigens family with specific fucosylation of the terminal end of carbohydrate structure catalyzed by the α1,2-fucosyltransferase [1,2]. The expression of Lewis y antigen primarily occurs during the embryogenesis period. Under physiologic conditions, its expression in adults is limited on the surface of granulocytes and epithelium [3]. However, elevated expression of Lewis y has been found in 70-90% of the human carcinomas of epithelial cell origin, including breast, ovary, prostate, colon cancers, and the high expression level is correlated to the tumor's pathological staging and prognosis [4-6]. It has been reported that the Lewis y antigen was expressed on a number of different molecular carriers, including 2 major ovarian cancer antigens (CA_125 _and MUC-1), suggesting the high incidence of Lewis y in ovarian cancer [7].

We have established the stable ovarian cancer cell line with high expression of Lewis y, RMG-I-H, through gene transfection technique to introduce the gene of human α1,2-fucosyltransferase (α1,2-FT) into the ovarian cancer cell line RMG-I in our previous works. We found that the RMG-I-H cells become highly tolerant to the anti-tumor drugs, 5-fluorouracil, carboplatin [8,9]. It suggested that the Lewis y antigen possessed the function of boosting the survival ability of ovarian cancer cells.

Activation of the PI3K pathway supports survival and proliferation of multiple cell lineages [10]. PI3K activation results in the localized increase of phosphorylated lipid second messengers at the plasma membrane. Key signaling intermediates are then recruited to the phosphorylated lipids via specialized lipid-binding domains, pleckstrin homology (PH) domains, and are themselves activated to initiate further signaling events [11,12]. One key effector molecule that is activated in this manner is the serine/threonine kinase Akt, which, when localized to products of PI3K activation, is able to phosphorylate multiple downstream substrates that mediate cell growth, survival, and metabolism [13-15]. Studies found that soluble Lewis y antigen (4A11) or its glucose analog, H-2 g, effect angiogenesis by inducing VEGF expression and signaling through PI3K pathway in the angiogenesis-rich rheumatoid arthritis [16].

Here we report that the cell proliferation of ovarian cancer cell line RMG-I sped up as the Lewis y antigen was increased. The phosphorylation level of Akt was apparently elevated in Lewis y-overexpressing cells. The inhibitor of PI3K, LY294002, dramatically inhibited the growth of Lewis y-overexpressing cells. Taken together, Lewis y antigen stimulates the growth of ovarian cancer cells through activating PI3K/Akt signal-transduction pathway. Potential treatment strategies through the inhibition of PI3K signaling pathway to target Lewis y signals may provide a useful approach for therapy of ovarian tumor growth.

## Methods

### Materials

The human ovarian cancer cell line, RMG-I, which was established from the tissues of human ovarian clear cell carcinoma, donated by Professor Iwamori Masao of Tokyo University of Japan. The following reagents were purchased from commercial sources: expression vector pcDNA3.1(-) and a TA cloning kit from Invitrogen (San Diego, CA, USA); E. coli (competent cells) JM109 from Toyobo (Tokyo, Japan); restriction endonucleases, BamHI, EcoRI, and G_418 _(geneticin) from Gibco; cell transfection and NucleoBond plasmid kits from GE Healthcare (Piscataway, NJ, USA); AmpliTaq Gold™ and a Bigdye™ terminator cycle sequencing ready reaction kit from Perkin-Elmer/Applied Biosystems (Foster City, CA, USA); DMEM and fetal bovine serum (FBS) from Hyclone (Logan, UT, USA); trypsin, ethylenediamine tetraacetic acid (EDTA), dimethyl sulfoxide (DMSO) and 3-(4,5-dimethylthiazol-2-yl)-2,5-diphenyltetrazolium bromide (MTT) from Amresco (Solon, OH, USA); SABC test kit from Boshide Biotech Co (Wuhan, China); α-L-fucosidase and methylene blue from Sigma (St. Louis, MO); PI3K inhibitor LY294002 from Promega (Madison, WI); primers and Reverse Transcription Polymerase Chain Reaction (RT-PCR) reagents are products of TaKaRa Biotechnology Co. Ltd (Dalian, China); mouse anti-human Lewis y monoclonal antibody from Abcam (UK); rabbit anti-human IgM monoclonal antibody, PCNA and β-actin from Santa Cruz Biotechnology (Santa Cruz, CA, USA); Akt and p-Akt from Cell Signaling Technology, Inc. (Beverly, MA, USA); protein content in cell lysates was measured by the BCA method (Beyotime, China).

### Cell culture

Cells were cultured in DMEM supplemented with 10% FBS at 37°C under 5% CO_2 _in humidified air.

### Construction of plasmid and generation of stably transfected cell lines

The human α1,2-fucosyltransferase gene (FUT-1) was amplified by PCR with human leukocyte genomic DNA as a template and primers according to the human FUT-1 gene sequence (GenBank Accession Number: M35531), sense primer, 5'-CATGTGGCTCCGGAGCCATCGTC-3', and antisense primer, 5'-GCTCTCAAGGCTTAGCCAATGTCC-3', under the following conditions: denaturation at 94°C for 9 min, followed by 25 cycles of 94°C, 1 min, 65°C, 1.5 min, and 72°C, 2 min, and then extension at 72°C for 10 min. The PCR products were ligated into the pCR2.1 vector to clone FUT-1 gene, and its DNA sequence was determined by means of the dideoxynucleotide chain-termination method with the BigDye terminator cycle sequenceing ready reaction kit and a DNA sequencer (ABI Genetic Analyzer; Perkin-Elmer/Applied Biosystems). Then the FUT-1 gene in pCR2.1 was cut out by digestion with restriction enzymes, BamHI and EcoRI, and ligated into the BamHI and EcoRI sites of the pcDNA3.1 vector (pcDNA3.1-hFUT). pcDNA3.1-hFUT and the vector alone were transfected into RMG-I cells with a vector transfection kit, according to the instructions for the kit to establish RMG-I-H and RMG-I-pcDNA3.1 cells, respectively. The resultant transfectants were initially selected by cultivation with medium containing an aminoglycoside antibiotic, G_418_, at 400 μg/ml concentration, and were maintained at 200 μg/ml for 15 days.

### Determination of α1,2-FT mRNA with semi-quantitative RT-PCR

Total RNA was extracted from the transfected and control cells using Trizol reagent. The cDNA was synthesized using Takara RNA PCR Kit and was used as a template for PCR analysis. The primer for α1,2-FT was F: 5'-GACTGTGGATCTGCCACCTG-3', R: 5'-GAAAGCTGTCTTGATGGATATGGAG-3' (fragment size, 131 bp). The primer for β-actin was F: 5'-GGACTTCGAGCAAGAGATGG-3', R: 5'-ACATCTGCTGGAAGGTGGAC-3' (fragment size, 404 bp). The cDNA was subjected to denaturation at 94°C for 5 min, followed by 30 cycles (94°C for 60 s, 65°C for 60 s, and 72°C for 60 s) of PCR and incubated at 5 minutes of 72°C. Then 10 μl of amplified products were detected by 2% agarose gel electrophoresis. The amplified DNA bands were scanned and analyzed with nih image software The quantitative data were obtained by the intensity ratios of α1,2-FT/β-actin band.

### Analysis the effect of Lewis y antigen on cell proliferation

Cells (2 × 10^3^/well) were planted in 96-well plates. MTT assay was used to detect cell proliferation for consecutive 7 days. In brief, MTT was added to the culture medium to yield a final MTT concentration of 0.5 mg/ml and the incubation was continued for 4 h at 37°C. The cell lysates were dissolved with DMSO at room temperature for 10 min. Results were obtained by measuring the absorbance at a wavelength of 490 nm. The test was repeated for three times.

### The removal of fucosyl residues on cell surface

The RMG-I-H and RMG-I (1 × 10^5^/ml) cells, were separately suspended in the solution of DMEM of pH 6.0, which included α-L-fucosidase (100 mU/ml). The laboratory requirement for removal of fucosyl residue followed the Sasak method [17], where the control sample was only added with DMEM of pH 6.0, excluding the addition of enzyme. The solution was incubated for 1 h at 37°C, and washed twice with DMEM of pH 7.25, before measurement. The enzyme concentration and incubation time were already determined before the experiment, and all fucosyl residues were mostly verified to be removed. The experimental group were named as RMG-I-H-A and RMG-I-A, respectively.

### Analysis the effect of α-L-fucosidase on cell proliferation

The cells before and after the process by α-L-fucosidase as above mentioned were seeded into 96-well plate at 3000 cells/well, and cell number was examined by MTT assay in triplicates for consecutive 7 days to detect cell proliferation. The test was repeated for three times.

### Colony formation test

Bottom agarose (0.7%) in DMEM was cast on 24-well plates. The cells before and after the process by α-L-fucosidase were mixed in 0.3% agarose in DMEM containing 10% FBS at 37°C and plated over the bottom agarose. The inoculated plates were incubated for 14 days and the number of cell clones with more than 50 cells was counted under microscope in each well (clone formation rate = number of clones in each dish/1000). Three reduplicate wells were used from each clone. Cell colonies were then fixed and stained with 0.5% methylene blue in ethanol. All colonies visible by eye were counted separately for each sample and evaluated their clone formation rates.

### Analysis the effect of anti-Lewis y antibody on cell proliferation

The RMG-I-H and RMG-I cells were separately added to 96-well plate at 3000 cells/well, after incubated for 2 h at 37°C in a humidifed atmosphere containing 5% CO_2_, Lewis y antibody (20 μg/ml) was added to wells as the experimental group, named as RMG-I-H-a and RMG-I-a, respectively; while rabbit anti-human IgM antibody of the same concentration was added as the control group, named as RMG-I-H-C and RMG-I-C, respectively. The cell number was examined by MTT assay in triplicates for consecutive 7 days to detect cell proliferation. The test was repeated for three times.

### Analysis the effects of the PI3K inhibitor LY294002 on cell proliferation

The RMG-I-H and RMG-I cells were seeded onto a 96-well culture plate at a density of 5000 cells/well in 100 μl of complete DMEM. On the second day of culture, the cells were then serum-deprived for 20 h prior to drug treatment. Quiescent cells were then exposed to media containing 10% FBS with LY294002 at a concentration of 3.125, 6.25, 12.5, 25 and 50 μM for 48 h. The cell number was examined by MTT assay in triplicates. The inhibitor was dissolved in DMSO to a stock concentration of 50 mM and DMSO served as a solvent control and did not affect cell proliferation. The assays were repeated three times, and the concentrations of LY294002 giving the IC50 were determined.

### Detection of the expression of Lewis y with immunocytochemical staining

The cells were seeded on the coverslips and fixed by 4% of paraformalclehyde, then stained according to the SABC test kit instructions. In brief, after blocking with goat serum for 1 h at 37°C, the mouse anti-human Lewis y antibody (1:100) was applied to incubate with the slide overnight at 4°C. Lewis y immunostaining was performed by avidin-biotin peroxidase complex kit and then photographed, where the existence of brownish yellow granules in cytoplasm and cell membrane would be considered as positive result.

### Western immunoblotting

After various treatments, cells were washed twice with ice-cold PBS, scraped in lysis buffer [50 mM Tris-HCl (pH 7.4), 150 mM NaCl, 0.5% NP40, 100 mM NaF, 200 μM Na_3_VO_4_, and 10 μg/ml each aprotinin, leupeptin, PMSF, and pepstatin], and incubated for 20 min at 4°C while rocking. Lysates were cleared by centrifugation (15 min at 13,000 rpm, 4°C). For immunoblot analysis, 50 μg of total protein were resolved by SDS-PAGE and transferred to poly(vinylidene difluoride) membranes. Membranes were blocked with TTBS [25 mM Tris-HCl, 150 mM NaCl (pH 7.5), and 0.1% Tween 20] containing 5% nonfat milk and incubated overnight at 4°C with primary antibody in TBST/1% nonfat milk. Blots were washed in TTBS and incubated with the appropriate horseradish peroxidaselinked IgG, and immunoreactive proteins were visualized with ECL detection system.

In the treatment of PI3K inhibitor LY294002 and anti-Lewis y antibody, the cells were subcultured for 72 h in serum-containing medium and then serum-deprived for 20 h, and then treated with 25 μM of LY294002 or treated with 20 μg/ml anti-Lewis y antibody for 24 h, and then the cells were harvested for immunoblot analysis.

### Statistical analysis

The SPSS 12.0 statistical analysis software was used, while the analysis of variance was employed. *p *< 0.05 was regarded as with statistical significance.

## Results

### Characterization of α1,2-FT-transfected cell lines

**Figure 1:**
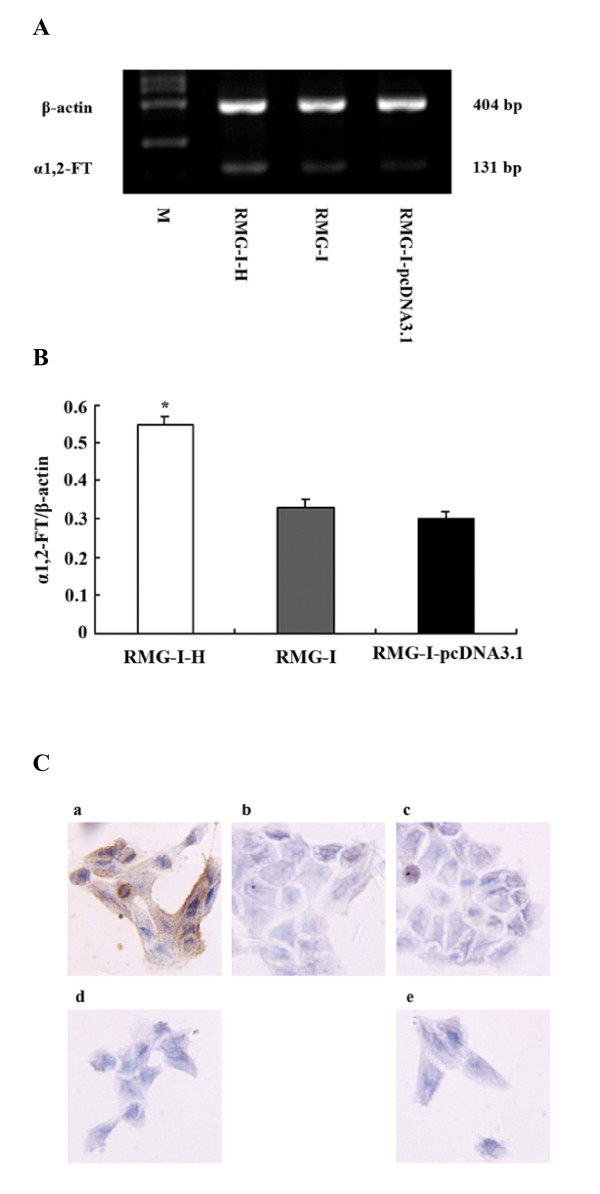
**Characterization of α1,2-FT-transfected cell lines**. (A) RT-PCR profiles of α1,2-FT mRNA in non- and α1,2-FT-transfected cells. M: DNA ladder marker (100-2000 bp). (B) Relative expression of α1,2-FT mRNA in non- and α1,2-FT-transfected cells (n = 3). The data was expressed as the intensity ratio of α1,2-FT to β-actin (Mean ± SD). * *p *< 0.01 compared to the control. "A" is the representative of three independent and reproducible experiments. (C) Immunohistochemical staining for Lewis y antigen. (a) RMG-I-H cells; (b) RMG-I-pcDNA3.1 cells; (c) RMG-I cells; (d) RMG-I-H-A cells; (e) RMG-I-A cells. Meanwhile, a, b and c represents cells without α-L-fucosidase treatmeant; d and e represents cells with α-L-fucosidase treatmeant.

The expressions of α1,2-FT mRNA in the pre- and post-transfection cell lines were measured by RT-PCR. Results showed that its expression of the post-transfection cell line RMG-I-H was significantly higher than those of RMG-I and RMG-I-pcDNA3.1 (Fig. 1A). Relative density analysis of α1,2-FT mRNA expression *vs*. their internal control β-actin expression indicated α1,2-FT mRNA expression in RMG-I-H was increased 2.07-fold with RMG-I and 2.23-fold with RMG-I-pcDNA3.1 (*p *< 0.01) (Fig. 1B). Furthermore, immunocytochemical staining revealed that the expression of Lewis y, the product of α1,2-FT, was also increased in RMG-I-H cells than that in RMG-I and RMG-I-pcDNA3.1 cells. The expression of Lewis y was mainly located on the cell surface (Fig. 1C).

### Lewis y overexpression promotes cell proliferation

**Figure 2:**
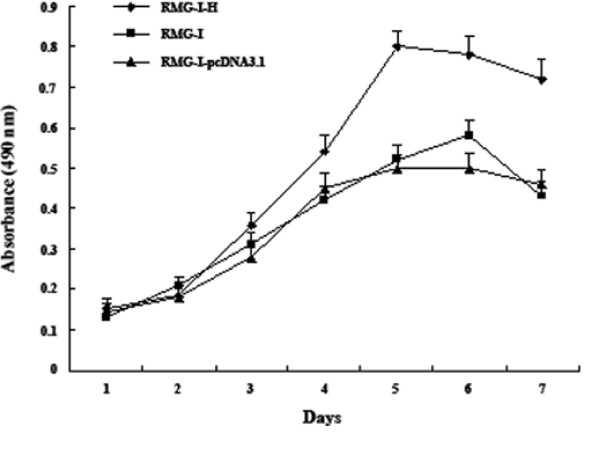
**The growth curves of each group of cells before and after the transfection**.

Lewis y overexpression significantly increased cell proliferation in culture as examined by MTT assay (Fig. 2). The proliferation rate of the post-transfection cells, RMG-I-H, was much higher than the non-transfected group and the group of transfected vector alone (*p *< 0.05). Also, there was no significance difference between the RMG-I and RMG-I-pcDNA3.1 (*p *> 0.05).

### α-L-fucosidase inhibits cell proliferation

Immunocytochemical staining technique was used to observe the expression of Lewis y in the cell lines before and after the process by α-L-fucosidase. As shown in Fig. 1C, the cytoplasm and cell membrane of RMG-I-H-A and RMG-I-A were without stains after the process by α-L-fucosidase, whereas, the cytoplasm and cell membrane of RMG-I-H did appear to have evenly distributed brownish yellow granules, while the RMG-I was very lightly stained.

**Figure 3:**
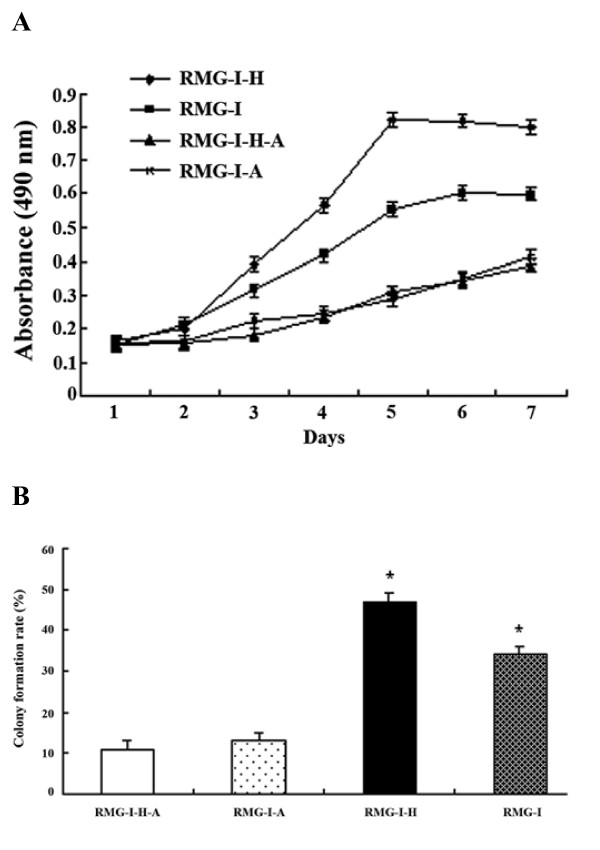
**Effects of α-L-fucosidase on the proliferation of the cells before and after the transfection**. (A) The cell growth curves of each group before and after the process by α-L-fucosidase (B) The colony formation rates of each group before and after the process by α-L-fucosidase. *p < 0.01 compared to the control.

The proliferation of the cells before and after the process by α-L-fucosidase was examined by MTT assay, as shown in Fig. 3A, the cell growth rates of the experimental group, RMG-I-H-A and RMG-I-A, were much lower than the control group, RMG-I-H and RMG-I, after the process by α-L-fucosidase (*p *< 0.01). There was no significant difference between RMG-I-H-A and RMG-I-A (*p *> 0.05), while the proliferation rate of RMG-I was still lower than that of RMG-I-H (*p *< 0.05). Colony formation test showed that the cells, after processed by α-L-fucosidase, were mostly single, the number of colony formation was much less and the size of colony was also smaller. The colony formation rates of RMG-I-H-A and RMG-I-A cells were 11% and 13%, respectively. While, the colony formation rates of RMG-I-H and RMG-I were 47% and 34%, respectively, which were significantly higher than those of the experimental group (*p *< 0.01) (Fig. 3B).

### Anti-Lewis y antibody inhibits the proliferation of Lewis y-overexpressing cells

**Figure 4:**
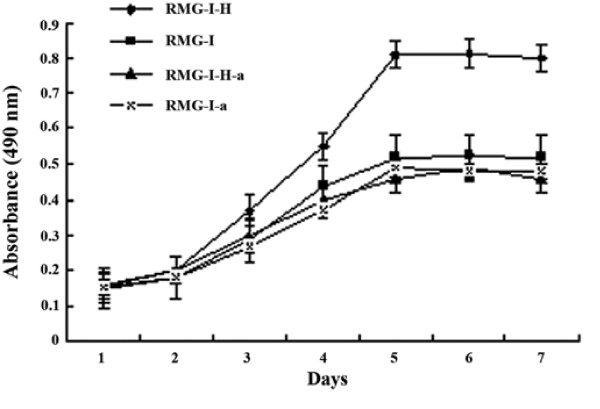
**The cell growth curves of each group before and after the process by anti-Lewis y antibody**.

Results in Fig. 4 showed that the cell growth of RMG-I-H cells was markedly inhibited by anti-Lewis y antibody, when compared with the control group RMG-I-H-C cells at the different time (*p *< 0.05). However, no significant difference in proliferation was found between RMG-I-a and RMG-I-C cells (*p *> 0.05). Meanwhile, the results in Fig. 4 also show that the proliferation rate of RMG-I was still lower than that of RMG-I-H (*p *< 0.05).

### LY294002 inhibits the proliferation of Lewis y-overexpressing cells

**Figure 5:**
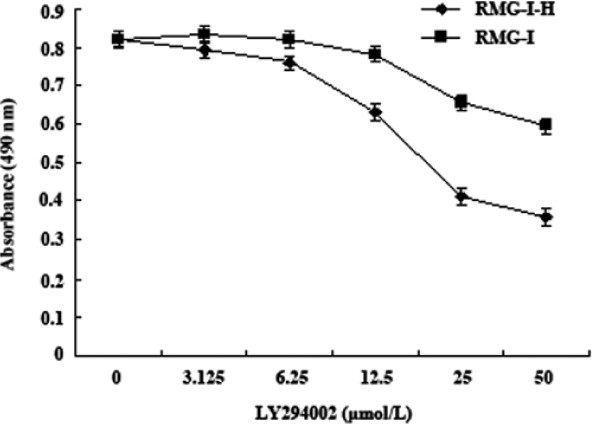
**The cell growth curves of each group before and after the process of LY294002**.

In order to investigate the mechanism of Lewis y-enhanced cell growth, we use the inhibitor of PI3K, LY294002, to treat the non- and α1,2-FT transfected cells, then the cell proliferation was observed. Results in Fig. 5 showed that when RMG-I-H cells were incubated with LY294002 at a concentration of 3.125, 6.25, 12.5, 25 and 50 μM for 48 h, respectively, the cell proliferation was inhibited, especially at the concentration of 25 and 50 μM, the number of proliferated cells was decreased significantly, the concentrations of LY294002 giving the half survival rates (IC50) were 23.18 ± 1.41 μM for RMG-I-H. In contrast, the proliferation of RMG-I cells was not significantly affected by treatment with various concentrations of LY294002.

### PI3K/Akt signaling is required for Lewis y-enhanced growth of RMG-I cells

**Figure 6:**
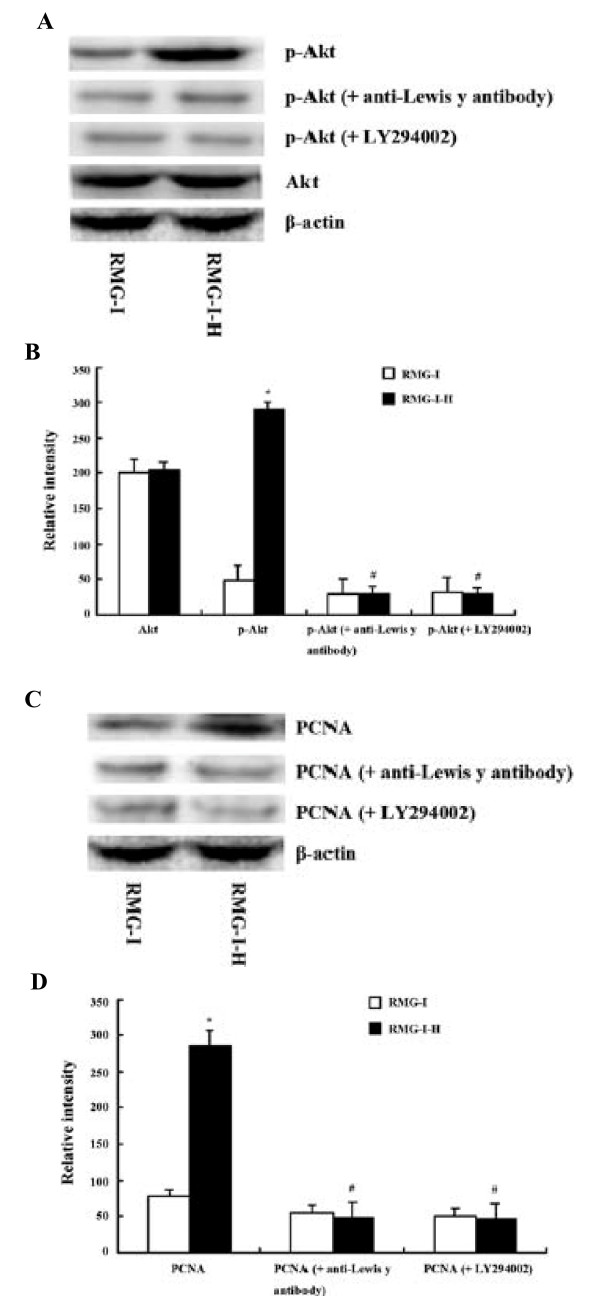
**PI3K/Akt signaling is required for Lewis y-enhanced growth of RMG-I cells**. (A) Western blot profiles of Akt and p-Akt in non- and α1,2-FT transfected cells, as well as in the absence and presence of anti-Lewis y antibody and LY294002. (B) Densitometric quantification of protein expression of A (n = 3). (C) Western blot profiles of PCNA in non- and α1,2-FT transfected cells, as well as in the absence and presence of anti-Lewis y antibody and LY294002. (D) Densitometric quantification of protein expression of C (n = 3).* *p *< 0.01 compared to RMG-I. # *p *< 0.01 compared to RMG-I-H cells without anti-Lewis y antibody or LY294002 treatment. "A" and "C" are the representative of three independent and reproducible experiments.

In grow factor signaling, activation of Akt has been implicated as a key step. As shown in Fig. 6A, B, expression of Akt protein was not obviously altered in Lewis y-overexpressing cells, but the relative phosphorylation of Akt (calculated from the ratio of the staining intensity of phophorylated protein to unphosphorylated protein after normalization with β-actin) was apparently upregulated to 5.37-fold of the non-transfection value in α1,2-FT transfected cells. Reults in Fig. 6A, B also show that when the two cell lines were treated by 20 μg/ml anti-Lewis y antibody or 25 μM LY294002 for 24 h (corresponding untreated cells were used as the control), phosphorylation of Akt was apparently decreased in non- and α1,2-FT transfected cells. By contrast, differences in phosphorylation intensity for Akt among non- and α1,2-FT transfected cell groups were attenuated in anti-Lewis y antibody- or LY294002-treated cells. When the cells were treated by anti-Lewis y antibody or LY294002, the rate of inhibition of phosphorylation was correlated with expression of Lewis y, which was Lewis y-highexpressing < Lewis y-lowexpressing cells.

PCNA is a commonly used marker to detect cell proliferation [18]. The difference in PCNA expression among these cells prepared as indicated above was also measured by western blotting. As shown in Fig. 6C, D, the expression of PCNA protein was significantly elevated to 3.64-fold of the non-transfection value in α1,2-FT transfected cells. Meanwhile, in the presence of anti-Lewis y antibody or LY294002, expression of PCNA, and the differences in its expression intensities among the two cell lines were also decreased, and the inhibition rate was also correlated with expression of Lewis y, which was Lewis y-highexpressing < Lewis y-lowexpressing cells.

## Discussion

Among the various post-translational modification reactions involving proteins, glycosylation is the most common, nearly 50% of all proteins are thought to be glycosylated [19]. Glycosylation reactions are catalyzed by the actions of glycosyltransferases, sugar chains being added to various complex carbohydrates [20]. An increasing body of evidence indicates that sugar chains in glycoproteins are involved in the regulation of cellular functions including cell-cell communication and signal transduction [21-23]. Research shows that 75% of ovarian cancers have varying degree of Lewis y overexpression, and increased expression is associated with poor prognosis of patients [24]. In our previous study, we introduced α1,2-FT gene into human ovarian cancer cell line RMG-I through gene transfection and established cell model overexpressing α1,2-FT gene and Lewis y [8]. Here by comparing cell proliferation status before and after transfection, we found that cell proliferation after gene transfection was accelerated. To further test the role of Lewis y in ovarian cancer cell proliferation, we treat Lewis y-overexpressing RMG-I-H ovarian cancer cells with α-L-fucosidase for the first time, which reducing the content of fucosylated antigens on cell surface. Through observing biological behaviors of cell before and after α-L-fucosidase treatment, we found the cell proliferation rate in transfected group was significantly higher than that of α-L-fucosidase-treatment group. Our preliminary study proved that the lactose type I chain family of the original RMG-I cells was primarily glycolipid, and they were Lc_4_Cer, Lewis a, and Lewis b, whereas, H-1 instead had the absolute domination in the successfully transfected cells. For the glycolipids of the lactose type II chain family, such as Lewis x, Lewis y, IV^3^NeuAc-nLc_4_Cer and NeuAc-Le^X^, their concentrations were over 0.01 μg per milliliter of dry cells; however, the glycolipids shown in the transfected RMG-I-H cells were Lewis x and Lewis y. 42.6% of Lewis x in the RMG-I-H was converted into Lewis y, which was in much higher percentage than the 3.2% of the original RMG-I cells. Although type I chain family H-1 had the absolute domination in the transfected RMG-I-H cells, its actual content was only 1/4 of the Lewis y [8]. These further proved that the changes of biological behaviors of RMG-I-H cells, such as enhancement of proliferation and growth, as well as the worsening in the severity of malignancy, all had to do with the increase in Lewis y antigen. Blocking experiments with Lewis y specific monoclonal antibody provided further evidence for its function.

The molecular mechanism by which Lewis y antigen causes the malignancy of ovarian cancer cell have not been completely understood. In previous studies, we tested the differences in oncogene expression before and after α1,2-FT gene transfection using gene chips technology. Results showed that: there were 88 differentially expressed genes after cell transfection, and altered genes mainly involved these genes regulating cell proliferation, signal transduction, transcription and so on [25]. Thus, it is possible that Lewis y may be an important component in signaling transduction pathway participating in signal transduction inside cell and further promoting proliferation of ovarian cancer cells. Studies found that anti-Lewis y antibodies (ABL364 and IGN311) blocked the activation of mitogen-activated protein kinase (MAPK) signaling pathway in A431 cells and prevented cell proliferation [26]. The MAPK signaling pathway has central roles in the regulation of cell survival and proliferation and our experimental results have further verified this conclusion. Our study found that the tyrosine phosphorylation level of MAPK after α1,2-FT gene transfection increased than that of before transfection in ovarian carcinoma-derived RMG-I cells (in press). In addition to MAPK pathway, the PI3K/Akt pathway is another critical pathway involved in cell survival and has been shown to be constitutivelsy active in ovarian cancer cell lines [27,28]. However, little is known about the relation of Lewis y and the PI3K/Akt pathway in the development and management of ovarian cancer. In an effort to understand the mechanism of action of Lewis y, we focused on investigating its effect on the PI3K/Akt pathway. In this study, we found the PI3K/Akt pathway was aberrantly activited by Lewis y antigen and PI3K/Akt pathway is necessary for Lewis y enhancing growth of RMG-I cells. It was verified by (1) increased tyrosine phosphorylation of Akt in α1,2-FT transfected cells. (2) blockage of cell surface Lewis y by anti-Lewis y antibody resulted in significant attenuation of the phosphorylation of Akt, as well as the difference in phosphorylation intensity among two cell lines. (3) in the presence of PI3K inhibitor LY294002, Lewis y no longer conferred a growth advantage in RMG-I-H cell. One of the crucial downstream targets of PI3K is the serine/threonine kinase Akt. Active Akt causes a variety of biological effects, including suppression of apoptosis by phosphorylation and inactivation of several targets along pro-apoptotic pathways. In particular, activated Akt is able to phosphorylate a variety of downstream substrates, e.g., Raf and I-K (a kinase that regulates the NF-κB transcription factor) [29]. A number of studies have demonstrated that the patients with increased p-Akt had a significant survival disadvantage compared to patients with lower Akt phosphorylation, and the patients with ovarian cancer suggested p-Akt overexpression as an independent prognostic indicator [30-32]. To our knowledge, this is the first report showing that overexpression of Lewis y antigen could significantly enhance proliferation of ovarian cancer cells through upregulating PI3K/Akt pathway.

Lewis y is mainly distributed at the plasma membrane of cancer cells [33], and carried by different glycolipids [34] and glycoproteins, such as CD44v6 [35], Muc6 [36] and epidermal growth factor receptor (EGFR) [37], which are related to carcinogenesis. Studies showed that changes in glycosyltransferase expression might affect structure of carbohydrate chains on cell surface receptors and therefore impacted the expression and function of those glycoprotein receptors [38,39]. It has been reported that transfection of the sense cDNA of *N*-acetylglucosaminyltransferase(GnT)-V, an enzyme associated with cancer progression and metastasis, into human H7721 hepatocarcinoma cells resulted in an increase in the level of GlcNAcβ1,6 Manα1,6-branch (GnT-V product) on the N-glycans of EGFR, this promoted the tyrosine autophosphorylation of EGFR [40]. From the above we speculate that there might be one possibility implicated in the mechanism that Lewis y antigen activates PI3K pathway. The over-expression of α1,2-FT cDNA results in the elevation of Lewis y content on some surface receptors, which might alter the comformation of the receptors, then promoting the signaling of the receptor and finally stimulating the proliferation of ovarian cancer cells. Our studies have found that the total amount of surface Lewis y as well as the Lewis y content on some surface receptors were all increased, and Lewis y expression on EGFR was very high on α1,2-FT-transfected cells (in press).

Cross-talk between the PI3K/Akt and the Raf/MEK/MAPK signaling pathways has been implied in human various malignant tumors, with some research stating that PI3K activity is essential for induction of Raf/MEK/MAPK activity [41,42]. Additional studies suggest that the PI3K/Akt pathway enhances and/or synergizes with Raf/MEK/MAPK signaling to provide a more robust survival signal [43]. We speculate whether such cross-talk between the two pathways also exists in Lewis y-overexpressing ovarian cancer cells, and whether Lewis y is the key point for triggering or regulating this cross-talk, the detailed mechanism requires further study.

The changes in glycosyltransferase expression might affect the sugar chain heterogeneity and distribution, which may mask some tumor antigens, reduce the immunogenicity of tumor cells, and promote tumor cells immune evasion. It has been confirmed that under normal circumstances, T lymphocytes do not recognize Lewis y antigen [44]. This allows the evasion of tumor cells from the recognition and killing by the human immune system, in order to easily enter the lymph nodes to form metastasis. Other studies found a novel function for soluble Lewis y, that is inducing cytokine release, such as interleukin-6 (IL-6), through the Janus kinase 2 (JAK2) pathway [45,16]. We speculate that except for proliferation, Lewis y could also induce tumor cells immune evasion through activating PI3K/Akt signaling pathway, the detailed mechanism is being studied. Lewis y may participate in natural humoral immune response, antibodies are ideally suited for eradicating pathogens from bloodstream and early tissue invasion. With regard to cancer cells, passively administered and vaccine induced antibodies have accomplished this concept, limiting tumor cells and systemic or intraperitoneal micrometastases in a variety of preclinical models. Many protocols developing anti-Lewis y vaccines have been performed [46,47].

In summary, we showed that increased expression of Lewis y antigen plays an important role in promoting cell proliferation through activating PI3K/Akt signaling pathway in ovarian carcinoma-derived RMG-I cells. Inhibition of Lewis y expression may provide a new therapeutic approach for Lewis y positive ovarian cancer.

## Conclusions

Lewis y overexpression promotes the proliferation of ovarian carcinoma-derived RMG-I cells and the PI3K/Akt signaling pathway is necessary for Lewis y-enhanced growth of RMG-I cells. These results may contribute for the development of a novel therapeutic methodology to treat Lewis y positive cancers.

## Competing interests

The authors declare that they have no competing interests.

## Authors' contributions

JL carried out most parts of the experiment; YH, LZ, FL, DL, JC and SZ participated in the experiment; BL participated in the design of the study; YQ performed the statistical analysis; IM participated in its design and coordination and helped to draft the manuscript. All authors read and approved the final manuscript.
